# Affective responses after different intensities of exercise in patients with traumatic brain injury

**DOI:** 10.3389/fpsyg.2015.00839

**Published:** 2015-06-25

**Authors:** Patricia Rzezak, Luciana Caxa, Patricia Santolia, Hanna K. M. Antunes, Italo Suriano, Sérgio Tufik, Marco T. de Mello

**Affiliations:** ^1^Department of Psychobiology, Universidade Federal de São PauloSão Paulo, Brazil; ^2^Department of Neurosurgery, Universidade Federal de São PauloSão Paulo, Brazil

**Keywords:** traumatic brain injury, mood, anxiety, exercise

## Abstract

**Background:** Patients with traumatic brain injury (TBI) usually have mood and anxiety symptoms secondary to their brain injury. Exercise may be a cost-effective intervention for the regulation of the affective responses of this population. However, there are no studies evaluating the effects of exercise or the optimal intensity of exercise for this clinical group.

**Methods:** Twelve male patients with moderate or severe TBI [mean age of 31.83 and SD of 9.53] and 12 age- and gender-matched healthy volunteers [mean age of 30.58 and SD of 9.53] participated in two sessions of exercise of high and moderate-intensity. Anxiety and mood was evaluated, and subjective assessment of experience pre- and post-exercise was assessed. A mixed between and within-subjects general linear model (GLM) analysis was conducted to compare groups [TBI, control] over condition [baseline, session 1, session 2] allowing for group by condition interaction to be determined. Planned comparisons were also conducted to test study hypotheses.

**Results:** Although no group by condition interaction was observed, planned comparisons indicated that baseline differences between patients and controls in anxiety (Cohens’ *d* = 1.80), tension (*d* = 1.31), depression (*d* = 1.18), anger (*d* = 1.08), confusion (*d* = 1.70), psychological distress (*d* = 1.28), and physical symptoms (*d* = 1.42) disappear after one session of exercise, independently of the intensity of exercise.

**Conclusion:** A single-section of exercise, regardless of exercise intensity, had a positive effect on the affective responses of patients with TBI both by increasing positive valence feelings and decreasing negative ones. Exercise can be an easily accessible intervention that may alleviate depressive symptoms related to brain injury.

## Introduction

Traumatic brain injury (TBI) is defined by the Brain Injury Association of America (BIAA, 2011) as “an alteration in brain function, or other evidence of brain pathology, caused by an external force.” Patients with TBI have a greater risk of experiencing emotional distress and depression due to their acquired brain lesion (Rosenthal et al., 1998). In a study of 559 TBI patients followed for 1 year following trauma, 53% of the sample met the criteria for major depression (Bombardier et al., 2010). The practice of regular physical activities has an indisputable positive effect for several health conditions (Blair, 1994; Allen, 1996; Magnusson et al., 1998; Uusitupa et al., 2000), including mental health (Stephens, 1988; Ströhle et al., 2007; Deslandes et al., 2009). Nevertheless, the World Health Organization (World Health Organization [WHO], 2010) recommendation for 150 min moderate-intensity aerobic exercise per week is a difficult target to achieve for people with disabilities (Paluska and Schwenk, 2000). Yet, the experience of improved mood states following acute bouts of physical activity in patients with TBI could serve as an immediate psychological reward for continuing exercise, and also serve public campaigns that encourage this population to adhere to a regular exercise practice. Thus, we aimed to determine the effects of a single bout of aerobic exercise and to establish the ideal exercise intensity in patients with TBI.

Studies demonstrating the positive effects of regular exercise on mental health have focused on healthy participants as well as patients with psychiatric disorders. In a large cohort of 55,000 healthy volunteers, leisure-time physical activity alone was associated with improved mental health, including reduced anxiety and depression symptoms, even after controlling for confounding factors such as age, sex, sociodemographic status, and physical illness (Stephens, 1988). A more recent, longitudinal-prospective study (Ströhle et al., 2007) of 2,548 participants from the community followed over a 4-years period demonstrated that regular exercise was associated with decreased prevalence of any mental disorder. However, there is evidence that this positive relationship is more robust in patients with a diagnosis of major depression (Conn, 2010). A program of supervised exercise in clinically depressed participants may have the same beneficial effects as psychotropic medication, with a better prognosis at a 6 months follow-up assessment (Blumenthal et al., 1999; Babyak et al., 2000). There is also evidence that mood may improve rapidly after only a single session of exercise, as compared to psychotropic medication, for which 40% of patients fail to respond, even after fourth-line therapy (Wade et al., 2014).

Researchers have shown that acute exercise can lead to an immediate improvement in positive affect (Reed, 2005; Barnes et al., 2010) and reductions in negative affect (Reed, 2005). Anxiety is reduced following bouts of exercise even in participants with normal or elevated levels of anxiety. This reduction may be seen 5–15 min after the cessation of exercise and can remain decreased for 2–4 h (Raglin, 1997).

An important question is whether the intensity of the exercise moderates the affective response of the practitioner. Research examining affective responses during exercise in non-athlete participants identified a negative relationship between exercise intensity and affect, such that as exercise intensity increased above ventilatory threshold (i.e., high-intensity exercise), people tend to feel greater psychological distress (Blanchard et al., 2001), less enjoyment (Brewer et al., 2000; Kilpatrick et al., 2003) and higher feelings of displeasure (Hall et al., 2002) as compared to moderate-intensity aerobic activities.

There have been few attempts to evaluate the effects of exercise on the mood and anxiety of patients with TBI, and there is no consensus as to whether exercise improves affective responses in this population. A study that investigated the relationship between exercise level and depressive symptoms in a community sample of individuals with TBI showed that TBI patients who exercised were less depressed than those who did not exercise (Gordon et al., 1998). Another report demonstrated that patients with TBI displayed reduced depression severity scores after a 10-weeks exercise program (Wise et al., 2012). On the other hand, two randomized control trials did not find a significant relationship between an implemented exercise program and depression scores (Bateman et al., 2001; Hoffman et al., 2010). Nevertheless, several methodological differences make it difficult to compare these studies. The first study (Gordon et al., 1998) did not implement an exercise program in an experimental study to investigate the effect of exercise on the mood of patients with TBI. The second study only investigated the effects of exercise on depression severity and did not have a comparison group (Wise et al., 2012). Finally, despite having a randomized control trial design, the other studies had different comparison groups. The first of these (Bateman et al., 2001) compared patients in a 12-weeks exercise program to patients who were allocated to a relaxation condition. The other study compared patients who exercised for 10 weeks to patients who did not receive any intervention (Hoffman et al., 2010).

The present study aimed to determine if aerobic exercise has a positive impact on the affective responses of patients with mild to moderate TBI. We also investigated whether workload is associated with a positive or a negative effect on the mood and anxiety of patients with TBI. Finally, we sought to establish if the impact of exercise on mood and anxiety is similar for patients with TBI and healthy controls. We hypothesized that patients submitted to a moderate-intensity aerobic exercise will display improvement on depression/anxiety scores, while a high-intensity exercise protocol will decrease positive feelings and evaluation of wellbeing in patients with TBI. We also hypothesized that controls would not show differences in affective responses after different exercise intensities.

## Materials and Methods

The Research Ethics Committee of the Universidade Federal de São Paulo (CEP 1858/09) approved all methods and procedures in accordance with the Declaration of Helsinki. All volunteers signed a written informed consent. This protocol was also registered at ClinicalTrial.gov (# NCT01395472).

## Subjects

### Eligible Criteria

The study included male patients under treatment at the Neurology and Neurosurgery Department of the Universidade Federal de São Paulo, aged 18–55 years-old, with at least 5 years of formal education, with a diagnosis of mild to moderate closed head injury (TBI in which the skull and dura mater remain intact) occurring more than 6 months prior, but after the age of 17. TBI severity was determined by the patient’s neurologist according to the following criteria: Glasgow Coma Scale between 3 and 12 or loss of consciousness ranging from 20 min to 36 h and post-traumatic amnesia for more than a day. Finally, all patients were seizure free for 1 year prior to the commencement of the study. A significant other accompanied all patients.

Any patient in acute phase of recovery (e.g., mental confusion), suffering post-traumatic amnesia, with a previous history of drug or alcohol abuse, brain lesions, or neurological or psychiatric disorders was excluded from the study. Additionally, patients who could not verbally express meaningful ideas clearly as determined by a neuropsychologist (P.R.) in a comprehensive neuropsychological evaluation or had compromised motor skills that could impede exercise in a recumbent cycle-ergometer were not included. Patients who had received psychiatric or psychological treatment for mood or anxiety disorders were also not included in the study.

Participants were first selected by the patient’s neurologist (I.S.) who was responsible for evaluating: the type (open or closed), the severity (mild, moderate or severe) and the phase of recovery (acute or chronic) of TBI. The same physician attested that exercise would not be dangerous for patient’s health. After initial selection, patients were interviewed by a neuropsychologist (P.R.) who guaranteed that patients met all eligible criteria for participating in the study.

The control group comprised of sedentary healthy male volunteers (not exercising regularly for at least 2 months before participation in the study), aged 18–55 years old. Subjects were selected using the following criteria: must have at least 5 years of formal education; sedentary lifestyle (i.e., no habitual exercise); no clinical symptoms or indicators of cardiovascular disease; no medication that could alter cardiovascular and cognitive function; no psychotropic drug use or any pharmaceutical drug for which exercise is a contraindication, or that may negatively influence cognitive function; and no recent surgical intervention. Volunteers were recruited from the University and were either university staff or post-graduate students.

Twenty-five patients were contacted between 2009 and 2011. Of these, 21 fulfilled the inclusion and exclusion criteria. Five patients dropped out before finishing the three sessions required (cardiovascular examination, high-intensity, and moderate-intensity physical activities), three patients were not allowed to participate in the study after the rest and effort electrocardiogram, and one patient had a severe aphasia. Therefore, only 12 patients completed the three sessions required in this study protocol and their data are presented in the present study. The Control group also comprised 12 age and sex-matched volunteers.

In the evaluated sample, the TBIs were due to vehicular accidents (58.33%), falls (30%), and injured in a physical fight (8.33%). The mean age of the injury was 3.56 years (SD ± 4.13; median = 2.58).

**Table 1** provides further information on participant characteristics, including cardiovascular history as wells as comparison between the patients and controls.

**Table 1:** Demographic and cardiovascular data for traumatic brain injury [TBI] patients and controls [mean (SD)].

	TBI Patients	Controls	*t*	*p*
Age (years)	31.83 ± 9.53	30.58 ± 9.53	0.35	0.727
Height (cm)	173.75 ± 9.00	178.00 ± 7.15	-1.28	0.213
Weight (kg)	74.36 ± 16.85	80.31 ± 9.67	-1.05	0.304
BMI (kg/m^2^)	24.48 ± 4.24	25.29 ± 2.28	-0.58	0.566
**Ergospirometry**				
VO_2_ peak (ml/kg/min^-1^)	33.18 ± 14.37	40.02 ± 5.30	-1.55	0.136
HR_max_ (bpm)	157.58 ± 15.34	175.67 ± 10.59	-3.36	0.003^∗^
HR_max_ achieved (%)	83.91 ± 9.15	92.72 ± 4.35	-3.01	0.008^∗^
VE max	75.82 ± 18.73	133.62 ± 44.45	-4.15	0.000^∗^
Work-load max (W)	145.83 ± 26.10	221.67 ± 38.34	-5.66	0.000^∗^
Work-load VT-1 (W)	86.67 ± 21.46	129.17 ± 31.18	-3.89	0.000^∗^

*cm, centimeters; kg, kilograms; m, meters; ml, milliliters; min, minutes; bpm, beats per minute; HR_max_, maximal heart rate; W, watts; VT-1, ventilatory threshold; VE max, maximal ventilation. ^∗^p < 0.05*.

### Procedures

#### Exercise

First, the participants completed an examination of cardiovascular responses to dynamic and static effort to evaluate cardiorespiratory aptitude. Those who did not show signs of harm after exercise completed two distinct exercise protocols: (1) progressive cycling to voluntary physical exhaustion (VPE; i.e., high-intensity exercise) and (2) 30 min of constant workload at a ventilatory threshold of 1 (CW; i.e., moderate-intensity exercise). As is further explained below, the physical workload of the CW session is based on the VPE session; the order of the sessions was therefore fixed.

The intervals between protocols 1 and 2 were a minimum of 1 week and a maximum of 2 weeks.

The tests were conducted in an acclimatized laboratory. In the VPE protocol, the subjects were equipped with a heart rate monitor (Polar, model FS1; Polar Kempele, Finland) and a mask (Hans Rudolph; Shawnee, KS, USA) before entering the adapted cycle-ergometer for inferior limbs (Load Angio with automatic stand, Netherlands). The warm-up was conducted for 3 min at a workload of 30 watts, and then the workloads were increased by 10 watts at 1-min intervals until VPE. Ventilation time course analysis, possible with ergospirometry test, reveals two disproportionate increases in the VO_2_ defining the first (VTI) and second (VTII) ventilatory thresholds. These disproportionate increases are related to exercise induced acidosis compensation and are related mostly to respiratory frequency increase during exercise (Wasserman, 1987; Wasserman and Koike, 1992).

The volunteers were asked to maintain a pedaling speed of 50 rpm during the whole protocol. Ergospirometry (COSMED, Quark PFT – Pulmonary Function Testing – FRC & DLCO, Italy) was used to measure the cardiorespiratory variables. These respiratory variables were maximal oxygen consumption (VO_2max_) in ventilatory thresholds I and II (VT-I and VT-II) and maximal heart rate (HRmax) and HR in VT-I and VT-II. To determine the oxygen consumption in VT-I and VT-II, the criteria described by Wasserman (1987) and Wasserman and Koike (1992) were followed. Before every test, the equipment was calibrated by trained personnel using a known gas concentration.

The CW protocol used the same equipment previously described. The subjects performed a 30-min constant workload in VT-I after the 3-min warm-up at predetermined 30-watt workloads. The affective response evaluations are referred to by the following abbreviations: before voluntary physical exhaustion (VPE-1), after voluntary physical exhaustion (VPE-2), before a constant workload at a ventilatory threshold of 1 (CW-1) and after a constant workload at a ventilatory threshold of 1 (CW-2).

#### Affective Response Evaluation

The psychological evaluation was performed in a quiet and acclimatized environment that was maintained at a temperature of 22 ± 2°C with 55% humidity. Two trained neuropsychologists conducted all evaluations in a standard sequence and with a maximum duration of 20 min. To minimize circadian variations, both sessions of exercise were performed at the same time of the day. Participants completed questionnaires to assess affective responses immediately before and after each exercise protocol. Mood, anxiety and well-being questionnaires were selected based on previous published studies that investigated the impact of exercise on affective responses (Blanchard et al., 2001; Bartholomew et al., 2005; Cassilhas et al., 2010). The following questionnaires were administered:

##### State–Trait Anxiety Inventory (STAI, Spielberger et al., 1980)

This questionnaire evaluated trait (how the person generally feels) and state (how the person is feeling at that moment) anxiety using 20 statements each. The volunteer was asked to consider a Likert-scale of 4 points. A higher total score relates to increased anxiety severity.

##### Brunel Mood Scale (BRUMS, Terry et al., 2003)

This scale was adapted from The Profile of Mood States (POMS). The BRUMS is a 24-item inventory with a Likert scale of 4 points that assesses the mood dimensions of Anger, Confusion, Depression, Fatigue, Tension, and Vigor.

##### Visual Analoge Mood Scale (VAMS, Bond and Lader, 1974)

This scale is a self-rating instrument used to measure mood. The participant is required to mark, according to their feelings, a 100 mm line that separates adjectives with opposite meanings. Each end of the scale is supposed to reflect extreme states of that condition. The VAMS score is determined by measuring the distance in millimeters from the left end of the card to the participant’s mark for each of the 16 adjectives. These items are organized into 4 subscales: ‘Anxiety,’ ‘Physical Sedation,’ ‘Psychological Sedation,’ and ‘Other Feelings.’

##### Subject Exercise Experience Scale (SEES, McAuley and Courneya, 1994)

This scale is a 12-item self-report measure of the subjective experiences that are unique to the exercise domain. These items are organized into three subscales: ‘Positive Well-Being,’ ‘Psychological Distress,’ and ‘Fatigue.’

### Data Analysis

A parametric analysis was performed after verifying the normality of the data through a Kolmogorov–Smirnov Test. Participant characteristics of demographic and physical data were compared using Student’s *t*-test. The research questions were addressed using a general linear model (GLM) to examine the main effect of Group (TBI, Controls) and Condition (baseline, VPE, CW), as well as the interaction between the two factors (Group × Condition). Baseline scores were calculated as the average across the two baseline sessions (VPE1 and CW1). Tukey’s HSD *post hoc* pairwise comparisons were performed after significant effects were obtained in GLM analysis. Planned comparisons were also performed according to the hypotheses of this study, which predict that mood and anxiety differences in baseline conditions between patients and controls would disappear after exercise (Tabachnick and Fidell, 2013). Therefore, a series of Student *t*-tests were run with between-group comparisons in each of the three measure points. According to Tabachnick and Fidell (2013), it is appropriate to conduct planned comparisons in relation to hypotheses even in the absence of a significant interaction in a GLM.

As a measure of the magnitude of change in mood and anxiety measures we calculated standardized indices of effect size (Cohen’s *d*: Cohen, 1977). Cohen (Cohen, 1977) defined effect sizes as “small, *d* = 0.2,” “medium, *d* = 0.5,” and “large, *d* = 0.8.” For statistical analysis, we used the Statistical Package for the Social Sciences, version 14.0 for Windows (SPSS Inc., Chicago, IL, USA), with level of significance set at α = 0.05.

## Results

### Demographic and Fitness Information

The patients and controls had similar age [*t*(22) = 0.35; *p* = 0.727], height [*t*(22) = -1.28; *p* = 0.213], weight [*t*(22) = -1.05; *p* = 0.304] and body mass index (BMI) [*t*(22) = -0.58; *p* = 0.566] and were thus considered comparable samples. In the ergospirometry test, although both patients and controls had a similar maximal oxygen volume [*t*(22) = -1.55; *p* = 0.136], the TBI patients had a lower maximal heart rate (total and percentage) [*t*(22) = -3.36; *p* = 0.003 and *t*(22) = -3.01; *p* = 0.008, respectively], maximal ventilation [*t*(22) = -4.15; *p* < 0.001], maximal workload [*t*(22) = -5.66; *p* < 0.001] and workload at VT-1 [*t*(22) = -3.89; *p* < 0.001]. Refer to **Table 1** for more information.

### Basal Affective Response Profile

Compared with healthy volunteers, patients had a higher trait and state anxiety in STAI [*t*(1,22) = 4.38; *p* < 0.001; Cohen’s *d* = 1.80 and *t*(1,22) = 4.31; *p* < 0.001; *d* = 1.77, respectively]; higher levels of tension [*t*(1,22) = 2.85; *p* = 0.009; *d* = 1.31], depression [*t*(1,22) = 2.36; *p* = 0.028; *d* = 1.18], anger [*t*(1,22) = 2.22; p:0.036; *d* = 1.08], confusion [*t*(1,22) = 3.16; *p* = 0.005; *d* = 1.70] and total score [*t*(1,22) = 2.71; *p* = 0.013; *d* = 1.23] of BRUMS; more psychological distress in SEES [*t*(1,22) = 2.45; *p* = 0.023; *d* = 1.28]; and more physical sedation [*t*(1,22) = 3.37; *p* = 0.003; *d* = 1.42] in VAMS. A comparison between the two groups’ basal affective responses are shown in **Tables 2** and **3**.

**Table 2:** Comparing the two modalities of exercise in the TBI patients and the healthy controls.

	TBI			Controls			Group	Condition	Interaction
	Basal	VPE Mean (SD)	CW Mean (SD)	Basal Mean (SD)	VPE Mean (SD)	CW Mean (SD)	*p*	*p*	*p*
STAI S	36.00 (7.53)	36.83 (9.66)	34.17 (7.74)	29.83 (5.06)	34.25 (5.56)	32.83 (6.28)	0.009^∗^	0.553	0.113
BRUMS TE	2.45 (2.16)	2.50 (2.15)	2.58 (2.61)	0.67 (1.15)	0.92 (1.62)	0.75 (1.36)	<0.001^∗^	0.936	0.958
BRUMS D	2.73 (3.82)	1.25 (2.90)	1.33 (2.02)	0.33 (0.65)	0.42 (0.79)	0.25 (0.87)	0.005^∗^	0.382	0.337
BRUMS A	0.55 (1.04)	0.67 (2.02)	0.08 (0.29)	0.17 (0.39)	0.17 (0.58)	0.00 (0.00)	0.046^∗^	0.231	0.388
BRUMS V	9.00 (3.63)	9.83 (3.33)	9.25 (3.55)	9.92 (2.61)	8.58 (3.00)	8.50 (3.29)	1.000	0.925	0.171
BRUMS F	1.64 (2.29)	5.25 (4.43)	4.92 (5.58)	1.83 (1.19)	6.83 (3.38)^∗^	4.58 (2.87)	0.550	<0.001^∗^	0.630
BRUMS C	2.36 (1.80)	2.08 (2.50)	1.75 (2.22)	0.17 (0.39)	0.75 (1.60)	0.42 (1.00)	<0.001^∗^	0.800	0.690
BRUMS T	0.82 (10.20)	2.42 (12.15)	1.42 (13.02)	-6.75 (3.98)	0.50 (7.98)	-2.50 (6.49)	0.031^∗^	0.328	0.404
SEES PW	15.36 (5.07)	15.27 (4.54)	16.75 (4.56)	17.08 (2.57)	14.83 (5.06)	15.00 (3.93)	0.950	0.648	0.302
SEES PD	6.64 (3.35)	8.00 (5.27)	6.50 (3.75)	4.17 (0.58)	6.75 (3.36)	5.58 (2.78)	*0.056*	0.175	0.646
SEES F	6.45 (2.81)	14.73 (6.56)	12.83 (9.88)	6.17 (2.66)	16.17 (5.70)^∗^	11.67 (4.08)	0.841	<0.001^∗^	0.694
VAMS A	25.62 (11.79)	23.87 (16.08)	20.30 (15.07)	14.12 (14.10)	30.23 (11.50)^∗^	23.43 (12.14)	0.911	*0.081*	0.132
VAMS PS	30.54 (9.78)	25.33 (15.96)	21.39 (16.68)	13.11 (6.89)	17.09 (13.59)	14.41 (10.52)	0.003^∗^	0.669	0.714
VAMS MS	16.28 (10.60)	11.33 (10.68)	16.81 (18.39)	20.50 (11.35)	13.68 (12.61)	16.52 (13.57)	0.501	0.333	0.838
VAMS O	18.09 (12.72)	16.40 (17.16)	11.59 (12.64)	7.91 (6.78)	12.56 (9.81)	10.07 (12.22)	0.144	0.581	0.698

*STAI T, Trait-State Anxiety Inventory – Trait Scale; STAI S, Trait-State Anxiety Inventory – State Scale; BRUMS, Brunel Mood Scale; BRUMS TE, Tension Scale; BRUMS D, Depression Scale; BRUMS A, Anger scale: BRUMS V, Vigor Scale; BRUMS F, Fatigue Scale; BRUMS C, Confusion Scale; BRUMS T, Total Scale; SEES, Subjective Exercise Experience Scale; SEES PW, Positive well-being Scale; SEES PD, Psychological Distress Scale; SEES F, Fatigue Scale; VAMS, Visual Analog Mood Scale; VAMS A, Anxiety Scale; VAMS PS, Physical Sedation Scale; VAMS MS, Mental Sedation Scale; VAMS O, Other Feelings Scale. ^∗^*p* < 0.05*.

**Table 3:** *Post-hoc* comparison between groups according to physical exertion.

	Baseline		VPE		CW	
	*p*-value	Cohens’ *d*	*p*-value	Cohens’ *d*	*p*-value	Cohens’ *d*
STAI S	<0.001^∗^	1.77	0.431	0.34	0.648	0.19
BRUMS TE	0.009^∗^	1.31	0.054	0.85	0.042^∗^	0.92
BRUMS D	0.028^∗^	1.18	0.347	0.45	0.101	0.75
BRUMS A	0.037^∗^	1.15	0.418	0.38	0.328	0.55
BRUMS V	0.110	-0.69	0.344	0.39	0.597	0.22
BRUMS F	0.677	-0.17	0.336	-0.41	0.856	0.08
BRUMS C	0.005^∗^	1.69	0.134	0.65	0.071	0.83
SEES PW	0.177	-0.60	0.829	0.09	0.325	0.41
SEES PD	0.023^∗^	1.28	0.501	0.29	0.504	0.21
SEES F	0.427	0.34	0.579	-0.23	0.709	0.17
VAMS A	0.095	0.71	0.277	-0.46	0.581	-0.23
VAMS PS	0.003^∗^	1.39	0.187	0.56	0.232	0.51
VAMS MS	0.340	-0.40	0.627	-0.20	0.965	0.002
VAMS O	0.084	0.75	0.508	0.28	0.767	0.12


*STAI S, Trait-state Anxiety Inventory – State Scale; BRUMS, Brunel Mood Scale; BRUMS TE, Tension Scale; BRUMS D, Depression Scale; BRUMS A, Anger Scale: BRUMS V, Vigor Scale; BRUMS F, Fatigue Scale; BRUMS C, Confusion Scale; SEES, Subjective Exercise Experience Scale; SEES PW, Positive Well-Being Scale; SEES PD, Psychological Distress Scale; SEES F, Fatigue Scale; VAMS: Visual Analog Mood Scale; VAMS A, Anxiety Scale; VAMS PS, Physical Sedation Scale; VAMS MS, Mental Sedation Scale; VAMS O, Other Feelings Scale. ^∗^*p* < 0.05*.

### Comparison between Affective Responses of the Two Groups in Pre- and Post-Exercise

A GLM analysis of variance examined main effects of Group and Condition and their interaction. A significant effect of group was observed. Patients scored significantly higher on the following measures: STAI state [*F*(1,22) = 7.33; *p* = 0.009; *d* = 0.64]; BRUMS tension [*F*(1,22) = 15.92; *p* < 0.001; *d* = 1.00], depression [*F*(1,22) = 8.60; *p* = 0.005; *d* = 0.81], anger [*F*(1,22) = 4.14; *p* = 0.046; *d* = 0.56], confusion [*F*(1,22) = 13.65; *p* < 0.001; *d* = 0.94] and total score [*F*(1,22) = 4.85; *p* = 0.031; *d* = 0.54]; and VAMS physical symptoms [*F*(1,22) = 9.41; *p* = 0.003; *d* = 0.75]. A statistics trend was observed for the SEES psychological distress [*F*(1,22) = 3.79; *p* = 0.056; *d* = 0.46; **Table 2**].

A significant effect of condition was also observed for BRUMS fatigue [*F*(1,22) = 9.94; *p* < 0.001] and SEES fatigue [*F*(1,22) = 14.33; *p* < 0.001]. A statistical trend was observed for VAMS anxiety [*F*(1,22) = 2.62; *p* = 0.081 ; **Table 3**). *Post hoc* analysis revealed differences in basal responses and VPE [*t*(1,22) = 4.34; *p* < 0.001; *d* = -1.65] and CW [*t*(1,22) = 3.07; *p* = 0.009; *d* = -1.08] of BRUMS fatigue and in basal responses and VPE [*t*(1,22) = 5.26; *p* < 0.001; *d* = -1.95] and CW[*t*(1,22) = 3.46; *p* = 0.003; *d* = -1.10] of SEES fatigue.

**FIGURE 1:**
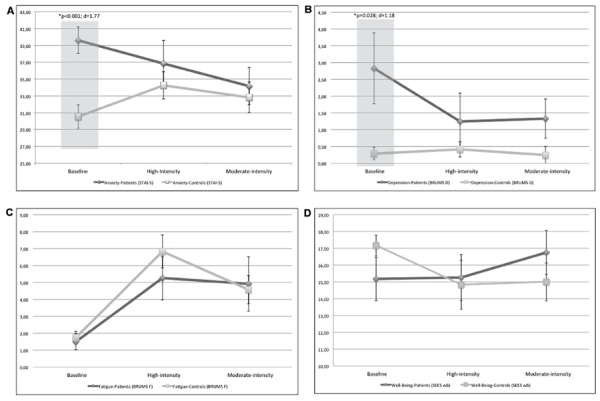
**Secondary exploratory omnibus ANOVA analysis comparing affective states of patients and controls in each of the three stages of the protocol; baseline, post high-intensity, and moderate-intensity exercises. (A,B)** Showing that baseline differences in anxiety and depression symptoms disappears after a session of exercise, regardless of the intensity of such activity. **(C,D)** Showing that states more related to physical evaluation (i.e., fatigue and well-being) remains similar between groups across the three measurements.

General linear model analysis of the interaction between Group and Condition was not statistically significant for any of the scales used. However, planned comparisons revealed baseline differences between groups in STAI state [*t*(1,22) = 4.31; *p* < 0.001; *d* = 1.77], BRUMS depression [*t*(1,22) = 2.36; *p* = 0.028; *d* = 1.18], anger [*t*(1,22) = 2.22; *p* = 0.037; *d* = 1.15], confusion [*t*(1,22) = 3.16; *p* = 0.005; *d* = 1.69], SEES psychological distress [*t*(1,22) = 2.45; *p* = 0.023; *d* = 1.28], VAMS physical symptoms [*t*(1,22) = 3.36; *p* = 0.003; *d* = 1.39], disappeared following both VPE and CWE sessions suggesting patient improvement following exercise. These data suggest that the mood, anxiety and psychological distress symptomatology present in patients before exercise decrease to healthy levels after exercise, despite exercise intensity. On the other hand, patients and controls respond similarly in fatigue and other well-being factors after exercise. To exemplify such response style, **Figure 1** shows slopes of depression, anxiety, fatigue and well-being scores for each sample and in each measure condition.

However, BRUMS tension scores, that were higher in patients in baseline evaluation [*t*(1,22) = 2.85; *p* = 0.009; *d* = 1.31], remained higher after CWE exercise [*t*(1,22) = 2.16; *p* = 0.042; *d* = 0.92] and tended to remain higher after VPE protocol [*t*(1,22) = 2.03; *p* = 0.054; *d* = 0.85].

## Discussion

The aim of this study was to evaluate the effects of exercise on the affective responses of patients with TBI and to determine whether the intensity of this exercise differentially impacts on negative and positive affect. Our most striking finding is that following exercise, patients with TBI no longer reported significant differences relative to healthy controls on affective responses. Moreover, the two groups showed similar responses after a moderate and a high-intensity exercise, demonstrating that patients with TBI responded similarly to different exercise protocols. The major finding here – based on planned comparisons – is that patients displayed reductions in depression and anxiety symptoms comparable to controls after both exercise intensities.

To the best of our knowledge, this is the first study to evaluate the effects of a single session of exercise on the psychological responses of patients with TBI in addition of being the first to test the hypothesis that the intensity of exercise may play a pivotal role in the beneficial effects of this type of activity on the affective responses of these patients. It is still unclear how long a person should exercise in order to benefit from its effect. In the present study we tried to establish if the positive effect on mental health, already demonstrated in healthy individuals (Stephens, 1988; Ströhle et al., 2007; Conn, 2010), could be observed following only a single session of exercise.

Three groups have previously studied the impact of a longer exercise program on the mood and anxiety symptoms of brain injury patients. While Gordon et al. (1998) demonstrated that exercise in individuals with TBI was associated with elevated mood and perceptions of better health, both Bateman et al. (2001) and Hoffman et al. (2010) did not find a significant relationship between aerobic exercise training and psychological aspects. Our findings are in agreement with the suggestion that aerobic exercise can improve affective responses in patients with TBI. Nevertheless, it is important to notice differences in our methodologies that limit the comparison between studies. First, Bateman et al. (2001) used a sample of heterogeneous brain injury patients comprising traumatic and vascular brain injury subjects. Moreover, they included patients with a recent history of brain injury, disregarding that premature exercise, close to the time of injury, can have a detrimental effect on brain functioning (Griesbach et al., 2004) and because of that is a trouble time to determine the real origin of any relationship between exercise and mental disorders. Additionally, both studies (Bateman et al., 2001; Hoffman et al., 2010) asked the subjects to self-control the intensity of their exercise by checking their heart rate and adjusting their pace to stay within a range of 60–80% of the maximum heart rate. However, it is impossible to guarantee that subjects actually work out at the desired intensity because they may intentionally exercise below the stipulated heart rate at a more comfortable pace or because they could not self-control their heart rate due to cognitive disabilities. In the present study the intensity of exercise was fixed and stipulated according to measures obtained during the ergospirometric exam. Most importantly, we recruited patients with no depression complaints and the affective response to exercise might be different for people with or without depression.

Prior research in non-TBI populations has suggested that the positive effect of exercise on mood, particularly of a single bout of exercise, was significantly greater in those with more depressive symptoms (Lane and Lovejoy, 2001). In agreement with such hypothesis, our TBI sample, which had some degree of depression and anxiety feelings, as evidenced by their baseline scores on STAI, BRUMS, and VAMS scores, showed improvement after one session of exercise, even though they did not have psychiatric disorder diagnosis. Besides, our control group composed of healthy volunteers with no complaints of mood disorders and no signs of depression in basal BRUMS or VAMS scores, did not show improvement after exercise in either positive or negative valence affective responses.

Depression-related changes have been described as one of the most common psychiatric syndromes following TBI. Studies on consecutive samples have found prevalence rates of depression ranging from 9 to 36% (Jorge et al., 2004; Bryant et al., 2010). Our findings suggest that a single session of exercise could serve as an intervention to aid daily mood regulation in patients with TBI. Future studies, following patients with TBI during a program of regular exercise are necessary to explore the long-term effect of this type of activity on mood and anxiety symptoms of this population.

One may pose the question of why exercise could be related to an improvement on mood. Some studies have demonstrated that exercise is associated with increased activity in both noradrenergic and serotonergic systems, which in turn have been associated to depression (Dunn and Dishman, 1991; Meeusen and De Meirleir, 1995; Dishman, 1997). Besides, changes in neurotrophic factors also have been noted, particularly for brain-derived neurotrophic factor (BDNF). The resultant increases in BDNF are associated with increased activity in monoaminergic systems, such as growth and regeneration of serotonergic neurons (Altar, 1999). The effect of BDNF has been likened to that of antidepressant treatment.

The view that the workload may be a moderator for the effects of exercise has been widely tested in studies that evaluated the impact of exercise on cognitive functioning. Moderate exercise is thought to improve cognitive performance in healthy adults and the elderly, whereas high-intensity exercise may instead impair brain processing (Chmura et al., 1994; McMorris et al., 1999). We have previously shown, using part of this dataset that some cognitive functions of TBI patients improved both VPE-2 and constant moderate-intensity workload exercises, although this improvement was noted on specific cognitive abilities in each case (Rzezak et al., unpublished data).

In a critical review about the acute effects of exercise on mood, Yeung (1996) demonstrated that the literature does generally support the belief that there are such effects. More than 85% of reviewed studies found at least some degree of improved mood on a wide variety of measures following exercise despite a diversity of exercise modes, durations and intensities (Cassilhas et al., 2010). Nevertheless, in non-clinical samples, a single bout of moderate-intensity exercise has been shown to reduce transiently depressive symptoms and improve moods more consistently than intense exercise (Yeung, 1996; Ekkekakis, 2003). Hence, there is still no consensus regarding the effects of high-intensity exercise on the mood and anxiety of non-clinical subjects.

In fact, one would expect that the fatigue levels after exhaustive exercise would be greater than after moderate-intensity exercise, which was not observed in the present study. One possible explanation for this observation is that exercising in the recumbent position with a heavy workload for a long period of time (30 min) could increase the discomfort level as well as the risk for cramps, which in turn would bring a decrease in positive well being and would hinder the positive valence feelings associated with moderate-intensity exercise.

For this reason, the horizontal cycle-ergometer used in this study may have been a limitation. However, because patients with TBI may have difficulties in postural control and other motor disabilities secondary to the brain injury (Gowland and Gambarotto, 1994), we believe that a vertical cycle-ergometer could impose a threat to the patient’s health, and because our main purpose was to evaluate the affective responses after exercise in patients with TBI, it is reasonable that this data is relevant information. Future studies would benefit by comparing TBI patients’ performance after an exercise session and other types of intervention, such as a stretching session. Another limitation of this study was the lack of a structural psychiatric interview to better describe the psychiatric status of the patients and controls. Although not as sensitive as a psychiatric evaluation, we were able to predict the presence of depressive and anxiety symptoms through the STAI, BRUMS, and VAMS scores.

Another possible weakness of this study is it small sample size. The use of rigorous selection criteria in order to have a homogeneous sample of male patients with a similar age-range, severity of TBI, in a chronic stage of recovery who have had their accident in adulthood imposed an obstacle to acquire a larger sample. Nevertheless, this was a compromise necessary for a better interpretation of our data as each one of these variables could serve as confounders for the analysis. Hence, future studies with larger samples are needed to corroborate our findings.

Although these limitations must be taken into account in the interpretation of the data, our findings are an important step in the discussion of whether exercise has a positive impact on the affective responses of patients with TBI. As this is an exploratory study, being the first to investigate this issue in this clinical population, more research is needed to corroborate the present data.

## Conclusion

A single-section of exercise, regardless of exercise intensity, had a positive effect on the affective responses of patients with TBI both by increasing positive valence feelings (such as psychological well-being) and decreasing negative ones (such as depression and anxiety feelings). This is a relevant finding, as exercise can be an easily accessible intervention that could alleviate depressive symptoms related to brain injury. Moreover, the identification of the positive effects of a single-session of aerobic exercise on mood and anxiety symptoms of patients can encourage the practice of such activities in a more regular basis.

## Conflict of Interest Statement

The Associate Editor, Dr Andrew Kemp, declares that despite being affiliated with the same institution as the authors, the review process was handled objectively. The authors declare that the research was conducted in the absence of any commercial or financial relationships that could be construed as a potential conflict of interest.

## References

[B1] Allen J. (1996). Coronary risk factor modification in women after coronary artery bypass surgery. *Nurs. Res.* 45 260–265. 10.1097/00006199-199609000-00002 8831651

[B2] Altar C. A. (1999). Neurotrophins and depression. *Trends Pharmacol. Sci.* 20 59–61. 10.1016/S0165-6147(99)01309-7 10101965

[B3] Babyak M. Blumenthal J. A. Herman S. Khatri P. Doraiswamy M. Moore K. (2000). Exercise treatment for major depression: maintenance of therapeutic benefit at 10 months. *Psychosom. Med.* 62 633–638. 10.1097/00006842-200009000-00006 11020092

[B4] Barnes R. T. Coombes S. A. Armstrong N. B. Higgins T. J. Janelle C. M. (2010). Evaluating attentional and affective changes following an acute e. *J. Sports Sci.* 28 1065–1076. 10.1080/02640414.2010.489196 20686994 PMC3042253

[B5] Bartholomew J. B. Morrison D. Ciccolo J. R. (2005). Effects of acute exercise on mood and well-being in patients with major depressive disorder. *Med. Sci. Sports Exerc.* 37 2032–2037. 10.1249/01.mss.0000178101.78322.dd 16331126

[B6] Bateman A. Culpan F. J. Pickering A. D. Powell J. H. Scott O. M. Greenwood R. J. (2001). The effect of aerobic training on rehabilitation outcomes after recent severe brain injury: a randomized controlled evaluation. *Arch. Phys. Med. Rehabil.* 82 174–182. 10.1053/apmr.2001.19744 11239307

[B7] Blair S. N. (1994). “Physical activity, fitness and coronary heart disease,” in *Physical Activity, Fitness and Health: International Proceedings and Consensus Statement*, eds Bouchard C. Shephard R. J. Stephens T. (Champaign, IL: Human Kinetics), 579–590.

[B8] Blanchard C. M. Rodgers W. M. Spence J. C. Courneya K. S. (2001). Feeling state responses to acute exercise of high and low intensity. *J. Sci. Med. Sport* 4 30–38. 10.1016/S1440-2440(01)80005-0 11339491

[B9] Blumenthal J. A. Babyak M. A. Moore K. A. Craighead W. E. Herman S. Khatri P. (1999). Effects of exercise training on older patients with major depression. *Arch. Intern. Med.* 159 2349–2356. 10.1001/archinte.159.19.2349 10547175

[B10] Bombardier C. Fann J. Temkin N. Esselman P. Barber J. Dikmen S. (2010). Rates of major depressive disorder and clinical outcomes following traumatic brain injury. *JAMA* 303 1938–1945. 10.1001/jama.2010.599 20483970 PMC3090293

[B11] Bond A. Lader M. (1974). The use of analogue scales in rating subjective feelings. *British J. Psychol.* 47 211–218. 10.1111/j.2044-8341.1974.tb02285.x

[B12] Brain Injury Association of America [BIAA]. (2014). *Brain Injury Association of America. BIAA Adopts New TBI Definition.* Available at: http://www.biausa.org/announcements/biaa-adopts-new-tbi-definition. (Accessed September 24, 2014).

[B13] Brewer B. W. Manos T. M. McDevitt A. V. Cornelius A. E. Van Raalte J. L. (2000). The effect of adding lower intensity work on perceived aversiveness of exercise. *J. Sport Exerc. Psychol.* 22 119–130.

[B14] Bryant R. A. O’Donnell M. L. Creamer M. McFarlane A. C. Clark C. R. Silove D. (2010). The psychiatric sequelae of traumatic injury. *Am. J. Psychiatry* 167 312–320. 10.1176/appi.ajp.2009.09050617 20048022

[B15] Cassilhas R. C. Antunes H. K. Tufik S. de Mello M. T. (2010). Mood, anxiety, and serum IGF-1 in elderly men given 24 weeks of high resistance exercise. *Percept. Mot. Skills* 110 265–276. 10.2466/pms.110.1.265-276 20391891

[B16] Chmura J. Nazar K. Kaciuba-Uściłko H. (1994). Choice reaction time during graded exercise in relation to blood lactate and plasma catecholamine thresholds. *Int. J. Sports Med.* 15 172–176. 10.1055/s-2007-1021042 8063464

[B17] Cohen J. (1977). *Statistical Power Analysis for Behavioral Sciences*, 2nd Edn, New York: Academic Press.

[B18] Conn V. S. (2010). Depressive symptom outcomes of physical activity interventions: meta-analysis findings. *Ann. Behav. Med.* 39 128–138. 10.1007/s12160-010-9172-x 20422333 PMC2870716

[B19] Deslandes A. Moraes H. Ferreira C. Veiga H. Silveira H. Mouta R. (2009). Exercise and mental health: many reasons to move. *Neuropsychobiology* 59 191–198. 10.1159/000223730 19521110

[B20] Dishman R. K. (1997). Brain monoamines, exercise, and behavioral stress: animal models. *Med. Sci. Sports Exerc.* 29 63–74. 10.1097/00005768-199701000-00010 9000157

[B21] Dunn A. L. Dishman R. K. (1991). Exercise and the neurobiology of depression. *Exerc. Sport Sci. Rev.* 19 41–98. 10.1249/00003677-199101000-00002 1682151

[B22] Ekkekakis P. (2003). Pleasure and displeasure from the body: perspectives from exercise. *Cogn. Emot.* 17 213–239. 10.1080/02699930302292 29715726

[B23] Gordon W. A. Sliwinski M. Echo J. McLoughlin M. Sheerer M. S. Meili T. E. (1998). The benefits of exercise in individuals with traumatic brain injury: a retrospective study. *J. Head Trauma Rehabil.* 13 58–67. 10.1097/00001199-199808000-00006 9651240

[B24] Gowland C. Gambarotto C. A. (1994). “Assessment and treatment of physical impairments leading to disability after brain injury,” in *Brain Injury Rehabilitation: Clinical Considerations*, eds Finlayson M. A. Garner S. H. (Philadelphia: Williams & Wilkins), 102–123.

[B25] Griesbach G. S. Hovda D. A. Molteni R. Wu A. Gomez-Pinilla F. (2004). Voluntary exercise following traumatic brain injury: brain-derived neurotrophic factor up regulation and recovery of function. *Neuroscience* 125 129–139. 10.1016/j.neuroscience.2004.01.030 15051152

[B26] Hall E. E. Ekkekakis P. Petruzzello S. J. (2002) The affective beneficence of vigorous exercise revisited. *British J. Health Psychol.* 7 47–66. 10.1348/135910702169358 14596717

[B27] Hoffman J. M. Bell K. R. Powell J. M. Behr J. Dunn E. C. Dikmen S. (2010). A randomized controlled trial of exercise to improve mood after traumatic brain injury. *PM R* 2 911–919. 10.1016/j.pmrj.2010.06.008 20970760

[B28] Jorge R. E. Robinson R. G. Moser D. Tateno A. Crespo-Facorro B. Arndt S. (2004). Major depression following traumatic brain injury. *Arch. Gen. Psychiatry* 61 42–50. 10.1001/archpsyc.61.1.42 14706943

[B29] Kilpatrick M. Hebert E. Bartholomew J. Hollander D. Stromberg D. (2003). Effect of exertional trend during cycle ergometry on postexercise affect. *Res. Q. Exerc. Sport* 74 353–359. 10.1080/02701367.2003.10609103 14510303

[B30] Lane A. Lovejoy D. (2001). The effects of exercise on mood change: the moderating effect of depressed mood. *J. Sports Med. Phys. Fitness* 41 539–545. 11687775

[B31] Magnusson C. Baron J. Persson I. Wolk A. Bergström R. Trichopoulos D. (1998). Body size in different periods of life and breast cancer risk in post-menopausal women. *Int. J. Cancer* 76 29–34. 10.1002/(SICI)1097-0215(19980330)76:1<29::AID-IJC6>3.0.CO;2-# 9533758

[B32] McAuley E. Courneya K. (1994). The subjective exercise experiences scale (SEES): development and preliminary validation. *J. Sport Exerc. Psychol.* 16 163–177.

[B33] McMorris T. Myers S. MacGillivary W. W. Sexsmith J. R. Fallowfield J. Graydon J. (1999). Exercise, plasma catecholamine concentrations and decision-making performance of soccer players on a soccer-specific test. *J. Sports Sci.* 17 667–676. 10.1080/026404199365687 10487466

[B34] Meeusen R. De Meirleir K. (1995). Exercise and brain neurotransmission. *Sports Med.* 20 160–188. 10.2165/00007256-199520030-00004 8571000

[B35] Paluska S. A. Schwenk T. L. (2000). Physical activity and mental health: current concepts. *Sports Med.* 29 167–180. 10.2165/00007256-200029030-00003 10739267

[B36] Raglin J. S. (1997). “Anxiolytic effects of physical activity,” in *Physical Activity and Mental Health*, ed. Morgan W. P. (Washington, DC: Taylor & Francis), 107–126.

[B37] Reed J. (2005). “Acute physical activity and self-reported affect: a review,” in *Causes, Role and Influence of Mood States*, ed. Clark A. V. (Chicago, IL: Nova Science Publishers, Inc.), 91–113.

[B38] Rosenthal M. Christensen B. Ross T. (1998). Depression following traumatic brain injury. *Arch. Phys. Med. Rehabili.* 79 90–103. 10.1016/S0003-9993(98)90215-5 9440425

[B39] Spielberger C. D. Vagg P. R. Barker L. R. Donham G. W. Westberry L. G. (1980). “The factor structure of the State-Trait Anxiety Inventory,” in *Stress and Anxiety*, eds Sarason G. Spielberger C. D. (Washington: Hemisphere), 95–109.

[B40] Stephens T. (1988). Physical activity and mental health in the United States and Canada: evidence from four popular surveys. *Prev. Med.* 17 35–47. 10.1016/0091-7435(88)90070-9 3258986

[B41] Ströhle A. Höfler M. Pfister H. Müller A. G. Hoyer J. Wittchen H. U. (2007). Physical activity and prevalence and incidence of mental disorders in adolescents and young adults. *Psychol. Med.* 37 1657–1666. 10.1017/S003329170700089X 17579930

[B42] Tabachnick B. G. Fidell L. S. (2013). *Using Multivariate Statistics*, 6th Edn Boston: Pearson.

[B43] Terry P. C. Lane A. M. Fogarty G. J. (2003). Construct validity of the POMS-A for use with adults. *Psychol. Sports Exerc.* 4 125–139. 10.1016/S1469-0292(01)00035-8

[B44] Uusitupa M. Louheranta A. Lindström J. Valle T. Sundvall J. Eriksson J. (2000). The Finnish diabetes prevention study. *British J. Nutr.* 83 S137–S142. 10.1017/s0007114500001070 10889804

[B45] Wade R. L. Kindermann S. L. Hou Q. Thase M. E. (2014). Comparative assessment of adherence measures and resource use in SSRI/SNRI-treated patients with depression using second-generation antipsychotics or L-methylfolate as adjunctive therapy. *J. Manag. Care Pharm.* 20 76–85. 24372461 10.18553/jmcp.2014.20.1.76 PMC10438233

[B46] Wasserman K. (1987). Determinants and detection of anaerobic threshold and consequences of exercise above it. *Circulation* 76 29–39. 3315297

[B47] Wasserman K. Koike A. (1992). Is the anaerobic threshold truly anaerobic? *Chest* 101 211–218. 10.1378/chest.101.5_Supplement.211S 1576837

[B48] Wise E. K. Hoffman J. M. Powell J. M. Bombardier C. H. Bell K. R. (2012). Benefits of exercise maintenance after traumatic brain injury. *Arch. Phys. Med. Rehabil.* 93 1319–1323. 10.1016/j.apmr.2012.05.009 22840829

[B49] World Health Organization [WHO]. (2010). *Global Recommendations on Physical Activity for Health*. WHO Library Available at: http://whqlibdoc.who.int/publications/2010/9789241599979_eng.pdf (acessed June 15, 2015). 26180873

[B50] Yeung R. R. (1996). The acute effects of exercise on mood state. *J. Psychosom. Res.* 40 123–141. 10.1016/0022-3999(95)00554-4 8778396

